# Pharmacokinetic and Pharmacodynamic Integration and Modeling of Enrofloxacin in Swine for *Escherichia coli*

**DOI:** 10.3389/fmicb.2016.00036

**Published:** 2016-02-02

**Authors:** Jianyi Wang, Haihong Hao, Lingli Huang, Zhenli Liu, Dongmei Chen, Zonghui Yuan

**Affiliations:** ^1^National Reference Laboratory of Veterinary Drug Residues (HZAU) and MAO Key Laboratory for Detection of Veterinary Drug Residues, Huazhong Agricultural UniversityWuhan, China; ^2^MOA Laboratory for Risk Assessment of Quality and Safety of Livestock and Poultry Products, Huazhong Agricultural UniversityWuhan, China; ^3^Hubei Collaborative Innovation Center for Animal Nutrition and Feed Safety, Huazhong Agricultural UniversityWuhan, China

**Keywords:** enrofloxacin, *Escherichia coli*, pig, pharmacokinetic, pharmacodynamic, PK/PD modeling

## Abstract

The aim of this study was to optimize the dose regimens of enrofloxacin to reduce the development of fluoroquinolone resistance in *Escherichia coli* (*E.*coli) using pharmacokinetic/pharmacodynamic (PK/PD) modeling approach. The single dose (2.5 mg/kg body weight) of enrofloxacin was administered intramuscularly (IM) to the healthy pigs. Using cannulation, the pharmacokinetic properties, including peak concentration (*C*_max_), time to reach *C*_max_ (*T*_max_), and area under the curve (AUC), were determined in plasma and ileum content. The *C*_max_, *T*_max_, and AUC in the plasma were 1.09 ± 0.11 μg/mL, 1.27 ± 0.35 h, and 12.70 ± 2.72 μg·h/mL, respectively. While in ileum content, the *C*_max_, *T*_max_, and AUC were 7.07 ± 0.26 μg/mL, 5.54 ± 0.42 h, and 136.18 ± 12.50 μg·h/mL, respectively. Based on the minimum inhibitory concentration (MIC) data of 918 *E. coli* isolates, an *E. coli* O_101_/K_99_ strain (enrofloxacin MIC = 0.25 μg/mL) was selected for pharmacodynamic studies. The *in vitro* minimum bactericidal concentration (MBC), mutant prevention concentration (MPC), and *ex vivo* time-killing curves for enrofloxacin in ileum content were established against the selected *E. coli* O_101_/K_99_ strain. Integrating the *in vivo* pharmacokinetic data and *ex vivo* pharmacodynamic data, a sigmoid *E*_max_ (Hill) equation was established to provide values for ileum content of AUC_24h_/MIC producing, bactericidal activity (52.65 h), and virtual eradication of bacteria (78.06 h). A dosage regimen of 1.96 mg/kg every 12 h for 3 days should be sufficient in the treatment of *E. coli*.

## Introduction

The *Escherichia coli* was the most important pathogen for post-weaning diarrhea which can cause the increase of mortality, morbidity and raising cost of pigs.

Enrofloxacin is a third generation fluoroquinolone developed by Guangdong Haikang veterinary Ltd. in 1993 in China. As a broad-spectrum antimicrobial drug used in veterinary medicine, its principal applications have been for respiratory and alimentary tract infections caused by gram-negative bacteria (Giguère et al., 2013). With the widespread use of enrofloxacin in husbandry and clinical setting, drug-resistant *E. coli* strains have emerged. A recent report showed that about 40.5% of the clinical *E. coli* isolated from pigs in Xinjiang was resistant to enrofloxacin (Xia et al., 2012).

The development of resistance may largely due to the misuse of antimicrobial agents (Burgess, 1999). The pharmacokinetics and pharmacodynamics (PK/PD) modeling could provide optimal dosage strategies and prevent resistance development. PK/PD modeling is a key tool that can help identify the clinically relevant relationship between time and effect. This study investigated the PK and *ex vivo* PD activity of enrofloxacin by using ileum content and plasma obtained from healthy pigs.

Cannulation of pig ileum was first used for studying nutrient digestibility in 1973 (Holmes et al., 1974). The simple-T cannulation method, which is widely used, has many advantages over other cannulation procedures. In this method, a hollow cannula is surgically inserted 8–10 cm anterior into the ileocecal valve. Simple-T cannulation maintains a normal physiological state, which is advantageous for research involving the gastrointestinal tract (Wubben et al., 2001). It also enables convenient ileum content sampling at different time points, which allow direct prediction of drug concentration at the site of action.

The aims of the present study were (i) to monitor the pharmacokinetic properties of enrofloxacin in plasma and in ileum contents after intramuscular (IM) dosing, (ii) to detect the *in vitro* and *ex vivo* pharmacodynamic parameters of enrofloxacin against pig source *E. coli*, and (iii) to determine a rational dosage regimen for enrofloxacin against *E. coli* based on PK/PD modeling. It is proposed that this dosage regimen will provide maximal efficacy and minimal opportunity for the emergence of resistant *E. coli* (Aliabadi et al., 2003).

## Materials and methods

### Animals

The study was carried out in six healthy male (castrated) pigs, weighing 21–32 kg (mean = 27 kg and *SD* = 3.82) at an age of 6–7 weeks. The pigs were placed in separate pens. Pigs had free access to water and were fed antibiotic-free food twice daily. Pigs were allowed a 7-day acclimation period prior to the study. Animal housing was maintained at 25 ± 2°C and 45–65% relative humidity. All the animal experimental were approved by Laboratory Animal Use and Care Committee in Hubei Science and Technology Agency and performed according to the committee guidelines. The anesthetics and other techniques were used to reduce the pain and adverse effect of animal.

### Drug administration

The 50 mg/mL enrofloxacin solution purchased from Baytril (Bayer AG, Leverkussen, Danmark). The approved label dose (2.5 mg/kg) was intramuscularly administrated to one side of the neck of the pigs.

### Insertion of ileal cannulation and sampling procedures

The cannula was exteriorized on the right side of the pig between the last two ribs (Wubben et al., 2001). The anesthesia was administered intravenously at the dose of 15 mg/kg and maintained with ketamine. The cannula was composed of 10 cm of medical grade rubber plastic tubing (acetal homopolymer resin), with inner and outer diameters of 2 and 3 cm, respectively. The large diameter end of the tubing was hand-tooled to provide a flanged end with a concave inner surface to conform to the shape of the ileum. All unthreaded areas on the cannula were hand-finished for smooth the surfaces and edges where the cannula would contact tissues. Pigs were allowed to recover fully in the metabolism crates for 2 weeks and were provided supplemental heat from an infrared heat lamp.

Blood samples (10 mL) were collected in heparinized tubes from the jugular catheter at 0 (pretreatment), 5, 15, 30, and 45 min, and at 1, 2, 4, 6, 8, 10, 12, 24 h after enrofloxacin administration. Keep the collection tubes on ice for 30 min, and then centrifuged at 2500 g, 4°C for 10 min. The collected plasma samples were stored at −20°C until assayed.

Ileum contents were collected into tubes from the ileal cannulation at different time point (0, 15, 45 min, 1, 2, 4, 6, 8, 12, 24, and 48 h) after enrofloxacin administration. Samples were divided into two aliquots on ice and stored at −20°C, for subsequent PK/PD studies.

### Establish high-performance liquid chromatography (HPLC) for detecting drug concentration

Plasma and ileum contents concentrations of enrofloxacin and its metabolite ciprofloxacin were determined by using a Waters 2695 series reverse-phase HPLC as described previously (Janusch et al., 2014).

One milliliter acetonitrile was added to 0.5 mL plasma and the solution was vortexed for 3 min. Following centrifugation at 8000 g and 4°C for 10 min, the supernantant was evaporated to dryness at 50°C under nitrogen. The residue was reconstituted in 0.5 mL mobile phase and vortexed. A 40-μL aliquot of the reconstituted sample was injected into the HPLC system. An Agilent SB-C_18_ column (250 mm × 4.6 mm i.d., 5 μm) was used for separation. The 0.1% formic acid and 13% acetonitrile were used as mobile phase.

Two milliliters of 0.5 M EDTA at pH 7.0 and 15 mL of dichloromethane were added to 1 g ileum content. The sample was extracted in sealed 50 mL tubes by shaking for 10 min and then centrifuged at 3500 g. The organic phase was collected and the extraction repeated twice. Pool and dry the combined extracts under nitrogen at 50°C. Use 1 mL mobile phase (0.1% formic acid and 11.5% acetonitrile) to dissolve the samples. Maintain the column temperature at 30 ± 5°C. Set up the UV detector at a wavelength of 278 nm. The injection volume and flow rate were 40 μL and 1 mL/min, respectively.

The standard curves of enrofloxacin and ciprofloxacin were ranged from 0.04 to 1 μg/mL (mg) with *r*^2^ > 0.999 in plasma and ileum content. The limits of quantification (LOQ) were 0.04 μg/mL in plasma and 0.1 μg/mg in ileum content. The mean recovery of enrofloxacin and ciprofloxacin was >85% in plasma samples and >70% in ileum content. The intra- and interassay coefficients of variation were <9.6%. The specificity of the method was good for the target substances. There was no endogenous interference on chromatograms.

### Pharmacokinetic analysis

PK parameters for plasma and ileum content drug concentrations were performed by WinNonlin software (version 5.2.1, Pharsight Corporation, Mountain View, CA, USA). Drug concentrations were plotted on semi-logarithmic graphs to choose appropriate PK models. The most suitable compartmental model was evaluated following the minimum Akaike's information criterion (Yamaoka et al., 1978). The one-compartment model was the most appropriate model for all tested pigs. This model was used to compute several PK parameters, including *C*_max_, *T*_max_, AUC and so on. All data are presented as mean ± SD.

### Pharmacodynamics analysis

#### Bacterial strain

Nine hundred and eighteen *E. coli* strains were isolated from pigs. These isolates were collected between 2012 and 2013 from sources in Anhui, Henan, Jiangxi, Hubei, and Henan provinces. According to the MIC_90_ values of sensitive strains, an *E. coli* O_101_/K_99_ strain with MIC similar to MIC_90_, was used to study the antimicrobial activity of enrofloxacin *in vitro*. *E. coli* ATCC 25922 strain was used as reference strain for antibiotic susceptibility determination. These strains were stored at −80°C in our lab.

### Determination of MIC, MBC, and MPC *In vitro*

The minimal inhibitory concentration (MIC) was determined in both broth and ileum content by microdilution according to the CLSI guideline (CLSI, 2012). MIC was the lowest concentration of enrofloxacin where visible bacterial growth was inhibited after 24 hours' incubation.

To determine the minimal bactericidal concentration (MBC), 100 μL suspension from the MIC determination wells were successively 10 fold diluted in MH broth. The colony forming unit (cfu) of each dilution was counted by spreading 10 μL on MH agar plates after 24 hours' incubation at 37°C. The MBC was the lowest concentration of enrofloxacin which could reduce 99.9% bacterial density. The final result was expressed as mean of five independent experiments.

The 10^10^ cfu/mL of *E. coli* was prepared to determine the mutant prevention concentration (MPC) by the agar method (Zhao and Drlica, 2001). Bacterial suspensions were spread on the agars containing serial dilutions of enrofloxacin (1, 2, 4, 8, 16, and 32 MIC). After 96 hours' incubation at 37°C, the MPC can be defined as the lowest concentration which could totally inhibited bacterial growth under anaerobic conditions.

### *In vitro* and *Ex vivo* time-killing curves

For the *in vitro* time-killing curves, the tubes containing bacteria (10^6^ cfu/mL) and different concentrations of enrofloxacin (1/2 MIC–32 MIC) were co-incubated at 37°C. The colony forming units in the samples collected at different time point (0, 1, 2, 4, 8, 12, 18, and 24 h) was determined by bacteria agar counting.

For the *ex vivo* time-killing curves, the bacteria (10^6^ cfu/mL) were co-incubated with ileum content samples obtained from pigs at different time point after treated by enrofloxacin. The viable counts were determined at 0, 1, 2, 4, 8, 12, 18, and 24 h. The time-killing curves obtained with ileum content were analyzed with a PD model described by the following equation.

$$\frac{dB}{dt}=k_{net}\times \left(1-\frac{B}{B_{\max}}\right)\times B-\left(\frac{E_{\max}+C^{\gamma }}{EC_{50}^{\gamma }+C^{\gamma }}\right)\times B$$

Where *B* is the number of bacterial cell expressed as cfu/mL; *k*_*net*_ the net growth rate; *B*_max_ the maximum number of bacteria; *E*_max_ the maximum killing rate; *EC*_50_, the concentration to reach half of maximal killing rate; and γ, the steepness.

The *ex vivo* time-killing curve was fitted to this model with the hypothesis of a decrease in enrofloxacin concentration according to incubation time using the mlxplore software (version-1.1.0, Lixoft, Orsay, France).

### PK/PD integration

The AUC_24h_/MIC and *C*_max_/MIC were used as the combined PK/PD parameters. Using the following inhibitory sigmoid *E*_max_ model to integrate the *ex vivo* AUC_24h_/MIC ratio and bacteria count change (cfu/mL) in ileum content during 24 h incubation (Aliabadi and Lees, 2001; Aliabadi, 2002; Aliabadi et al., 2003). This model is described as follows:

$$E=E_{\max}-\frac{(E_{\max}-E_{0})\cdot C^{N}}{C^{N}+EC_{50}^{N}}$$

In the above formula, the E means effect of antimicrobial agent measured as log_10_ difference of bacterial number before and after 24 hours' incubation in the ileum content sample; E_0_ and *E*_max_ are the changes in log_10_ difference in bacterial count between 0 and 24 h in the control sample and in the enrofloxacin containing samples, respectively. *EC*_50_ is the AUC_24h_/MIC value which reached to 50% of the *E*_max_; C is the tested AUC_24h_/MIC ratio; and N is the Hill coefficient.

The *ex vivo* antibacterial effects of enrofloxacin administration were quantified into three levels including: (1) bacteriostatic action (no change in bacterial count, *E* = 0), (2) bactericidal action (99.9% reduction in bacterial count, *E* = −1), and (3) bacterial elimination (99.99 % reduction, *E* = −3) (Aliabadi and Lees, 2001; Aliabadi et al., 2003; Aliabadi, 2002; Aliabadi et al., 2003). The dose was calculated by the using the following formula.

$$DO=\frac{\left(AUC/MIC\right)\times MIC\times CL}{fu\times F}$$

where AUC/MIC is the targeted end point for optimal efficacy; MIC is minimum inhibitory concentration; CL is clearance per day; fu is free fraction of drug in plasma (ignore if minimal binding). In this study, the protein binding of enrofloxacin can be discounted because the ileum content enrofloxacin concentrations is higher than that in plasma. In other scientific literature (Nielsen and Gyrd-Hansen, 1997) the bioavailability (F) of enrofloxacin was 100%, so F may also be discounted.

To investigate the effect of different dosage regimens, the PD model describing bacterial growth rate as a function of enrofloxacin concentration was combined with the PK model and simulations were performed with mlxplore software (version-1.1.0, Lixoft, Orsay, France).

## Results

### Pharmacokinetics of enrofloxacin

**Figure 1:**
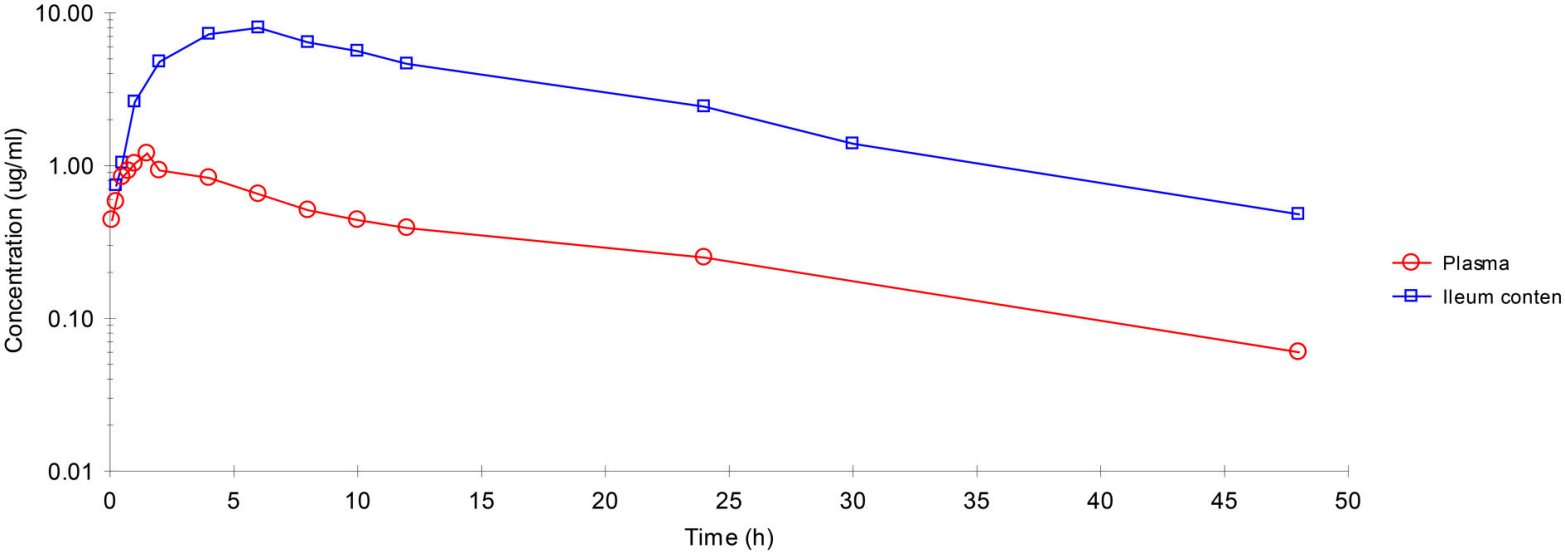
**Enrofloxacin concentration-time profiles plotted for plasma, ileum content after IM administration**. Values are mean ± SD (*n* = 6).

Plasma and ileum content concentrations of enrofloxacin IM administration are illustrated in Figure 1. The active metabolite ciprofloxacin was not found in any sample.

After IM dosing, the plasma concentration of enrofloxacin was best fitted with a mono-compartmental model (Table 1). Rapid absorption of enrofloxacin was indicated by T½abs = 0.28 h. *C*_max_ was 1.09 μg/mL, achieved at 1.27 h. The mean elimination half-life (T½el) was 6.69 h. AUC_24h_ was 12.7 μg·h/mL. The mean residence time (MRT) up to last was 8.48 h.

**Table 1:** **Pharmacokinetics of enrofloxacin in plasma and ileum content after IM administration (values are mean ± SD, *n* = 6)**.

**Variable (units)**	**Plasma**	**Ileum content**
AUC	12.70±2.72	136.18±12.50
T½abs	0.28±0.10	2.05±0.41
T½el	6.69±1.71	8.50±1.66
*T*_max_	1.27±0.35	5.54±0.42
*C*_max_	1.09±0.11	7.07±0.26
V/F	2.17±1.16	0.22±0.37
CL/F	0.20±1.08	0.072±0.18
MRT	8.48±0.67	14.38±0.03

AUC, Area under the concentration-time curve; T½abs, First order absorption rate constant; T½el, First order elimination rate constant; CL/F, Systemic clearance; V/F, Volume of distribution; MRT, Mean residence time.

After IM dosing, the ileum content concentration of enrofloxacin was also best fitted with a mono-compartmental model (Table 1). T½el was 8.50 h, showing slow elimination after IM administration. *C*_max_ was 7.07 μg/mL, achieved at 5.54 h. T½abs was 2.05 h. AUC_24h_ was 136.18 μg·h/mL. MRT was 14.38 h.

After IM administration, ileum content enrofloxacin concentrations were significantly higher than those in plasma.

### Pharmacodynamics

#### MIC, MBC, and MPC of enrofloxacin against *E. coli* strains

The MIC_50_ and MIC_90_ of 918 strains isolated from pigs in five provinces were 2 and 32 μg/mL, respectively. Using CLSI breakpoints, there were 178 susceptible *E. coli* isolates. The MIC_50_ and MIC_90_ of theses trains were 0.125 and 0.25 μg/mL, respectively (Table 2). According to the MIC_90_ values of sensitive strains, an *E. coli* O_101_/K_99_ strain with MIC similar to MIC_90_, was used to study the antimicrobial activity of enrofloxacin *in vitro*.

**Table 2:** **Susceptibilities (MIC_50_ and MIC_90_) of 918 *E. coli* strains isolated from pigs in Anhui, Henan, Jiangxi, Hubei, and Henan province, 2012–2013**.

**Antibiotic**	***E. coli* (*n* = 918)^a^**			***E. coli* (*n* = 178)^b^**		
	**MIC_50_ (μg/mL)**	**MIC_90_ (μg/mL)**	**Range (μg/mL)**	**MIC_50_ (μg/mL)**	**MIC_90_ (μg/mL)**	**Range (μg/mL)**
Enrofloxacin	2	32	0.015–128	0.125	0.25	0.015–0.5

a *n, total number of isolates studied*.

b *n, susceptible strains based on the CLSI breakpoints*.

MIC of enrofloxacin in MHB, plasma, and pig ileum content to *E. coli* O_101_/K_99_ strain were 0.25, 0.25, and 0.5 μg/mL, respectively. Corresponding MBC values were 0.5, 1, and 2 μg/mL, respectively. The MPC of enrofloxacin in MHB was 7.2 μg/mL (Table 3).

**Table 3:** **MIC, MBC, and MPC (μg/mL) of enrofloxacin against *E. coli* O_101_/K_99_**.

**Matrix**	**MIC (μg/mL)**	**MBC (μg/mL)**	**MPC (μg/mL)**
MHB	0.25	0.5	7.2
Plasma	0.25	1	–
Ileum content	0.5	2	–

### *In vitro* and *Ex vivo* antimicrobial activity

**Figure 2:**
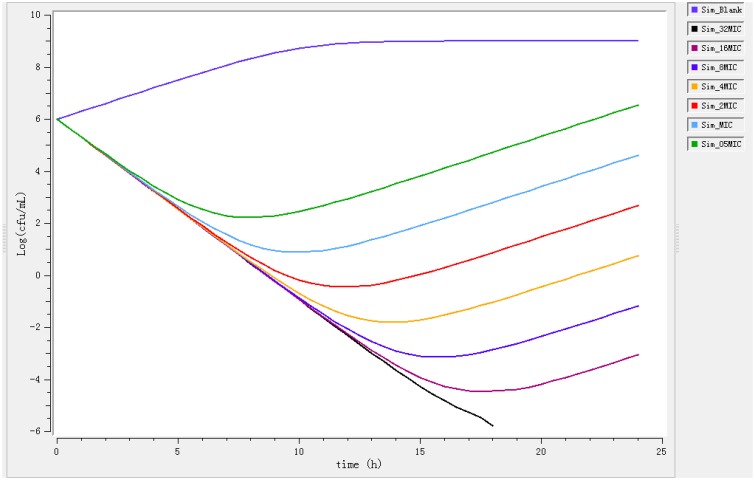
***In vitro* antibacterial activity of enrofloxacin against *Escherichia coli* in MHB**.

Time-killing curves obtained against *E. coli* O_101_/K_99_ for different concentrations of enrofloxacin expressed as multiples of MIC are showed in Figure 2. The curves are characteristics of concentration-dependent antibiotic activity. The net growth rate is lower for concentrations below 2 MIC. Meanwhile, the bactericidal activity increased with increasing concentration of enrofloxacin.

**Figure 3:**
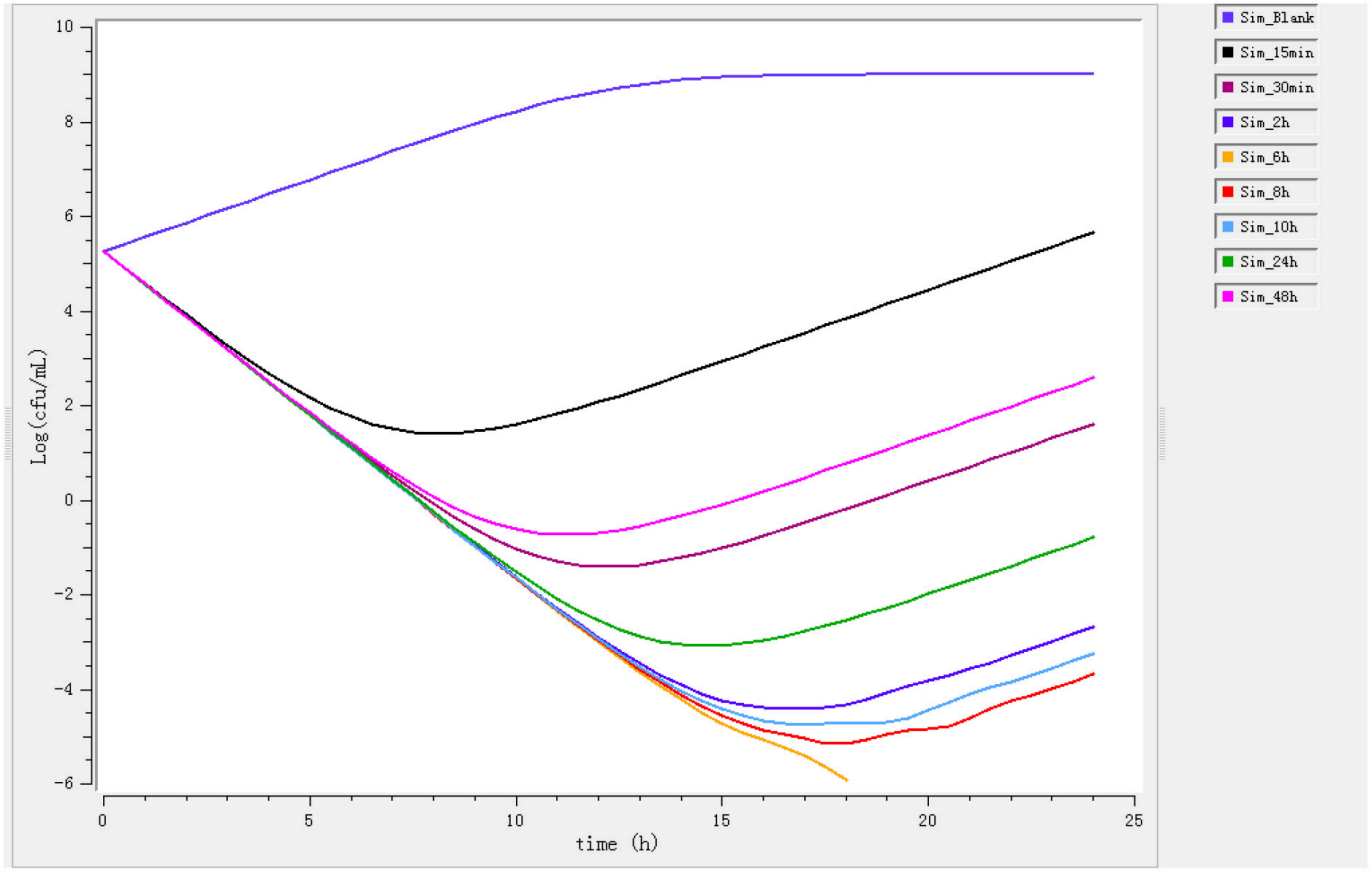
***Ex vivo* antibacterial activity of enrofloxacin in ileum content of swine against *Escherichia coli* after intramuscular administration (*n* = 6)**.

Ileum content samples from six pigs that had been administered enrofloxacin intramuscular collected at different time points were used to determine *ex vivo* killing rate. The results show that at the highest concentration (at 6 h), the number of bacteria decreased drastically (Figure 3). The growth is observed for ileum content samples collected before 0.5 h and a net killing rate is obtained with samples collected after 1 h. In the data set with ileum content, a decrease in bacteria number was shown in the *ex vivo* time-killing curves, however, a regrowth was observed during the 24 hours' incubation.

After fitting, the net killing rate was 0.69 h^−1^, *k*_net_ = 0.35 h^−1^, the maximum number of bacteria *B*_max_ was (10E8.16), *E*_max_ = 2.3 h^−1^, *EC*_50_ = 0.35 μg/mL, γ = 0.70 and enrofloxacin decrease rate was 0.26 h^−1^.

### PK/PD integration

The PK/PD indices *C*_max_/MIC, AUC_24h_/MIC, *C*_max_/MPC, and AUC_24h_/MPC of enrofloxacin against *E. coli* were integrated using the PK parameters and MIC data in ileum content (Table 4). The AUC_24h_ obtained in this study was 136.18 ± 12.5 μg·h/mL following a single dose IM administration of enrofloxacin. The mean AUC_24h_/MIC ratio was 272.4 h. The *C*_max_/MIC ratio was 14.1. The mean values for *C*_max_/MPC and AUC_24_/MPC were 0.98 and 18.9, respectively.

**Table 4:** **PK/PD integration parameters for enrofloxacin in ileum content after intramuscular injection of enrofloxacin at a dose rate of 2.5 mg/kg (*n* = 6)**.

**Parameter**	**Units**	**Mean ± SD**
*C*_max_/MIC	–	14.14
*C*_max_/MPC	–	1
AUC_24h_/MIC	–	272.36
AUC_24h_/MPC	–	18.9
T_>MIC_	h	33.54 ± 1.47
T_>MBC_	h	16.89 ± 2.56

### PK/PD modeling

In ileum content, the inhibitory sigmoid *E*_max_ model perfectly described the relationship between antimicrobial efficacy of enrofloxacin and the PK/PD parameter of AUC_24h_/MIC ratio. Table 5 showed that the parameters obtained N, E_0_, *E*_max_ and AUC_24h_/MIC values which respectively matched three level of antibacterial activity. Ileum content values of AUC_24h_/MIC for bacteriostatic activity, bactericidal action and virtual eradication were 21.37, 52.65, and 78.06, respectively.

**Table 5:** **PK/PD Modeling of *ex vivo* data after administration of enrofloxacin in pigs**.

**Parameter**	**Units**	**Mean ± SD**
*E*_max_	h·μg/mL	2.23 ± 0.50
EC_50_	μg/mL	34.17 ± 3.26
E_0_	μg/mL	−5.34±0.13
N	μg/mL	1.86 ± 0.39
*E*_max_ − E_0_	–	7.57 ± 0.98
Bacteriostatic (*E* = 0)	–	21.37 ± 1.85
Bactericidal (*E* = −3)	h	52.65 ± 3.78
Eradication (*E* = −4)	h	78.06 ± 2.41

### Estimation of dose

Based on the bactericidal AUC_24_/MIC ratio (52.65 h) and MIC of 0.5 μg/mL in ileum content, an intramuscular dosage of 1.96 mg/kg/day of enrofloxacin was calculated for bactericidal activity against *E. coli*. Moreover, given an AUC_24h_/MIC ratio for bacterial eradication (78.06 h), a dosage of 2.8 mg/kg/day is recommended to achieve virtual elimination of *E. coli*.

### Assessment of dose

**Figure 4:**
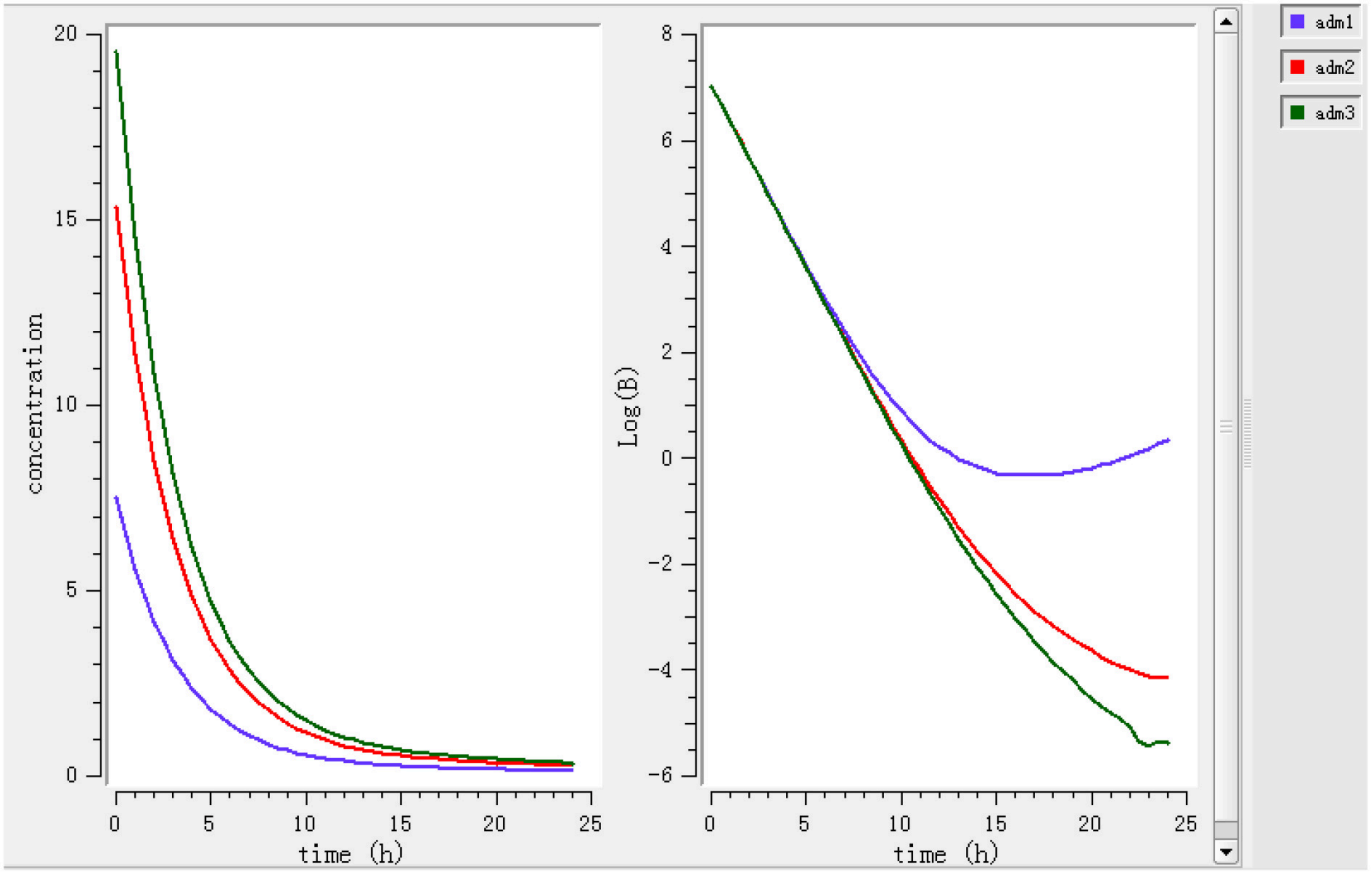
**Simulate the effect of different doses (0.78, 1.96, 2.8 mg/kg)**. The effects of different doses were observed on bacteria and its elimination.

Using a PK/PD model with the PD parameters derived from *ex vivo* analysis, actions of different doses (0.78, 1.96, 2.8 mg/kg) were simulated (Figure 4). According to these figures, a dose of 0.78 mg/kg is not sufficient to reduce the bacterial load while a dose of 1.96 mg/kg leads to a net reduction by a factor of 10 after 12 h.

**Figure 5:**
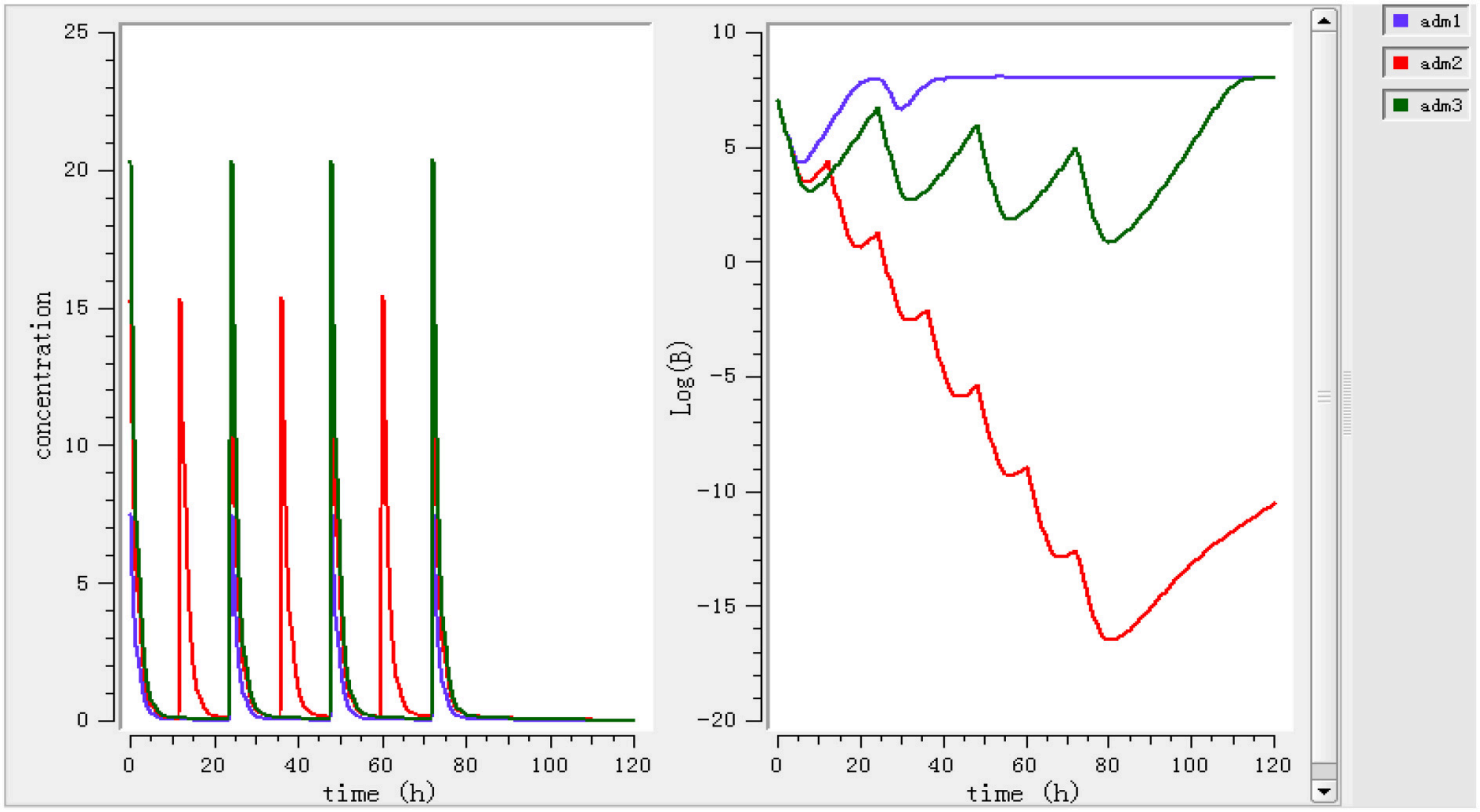
**Simulate different dosage regimen (0.78 mg/kg every 24 h, 1.96 mg/kg every 12 h and 2.8 mg/kg every 24 h)**. The different doses were simulated for different intervals of time to find the efficient dose and dose intervals.

Different dosage regimens for 3 days of treatment (0.78 mg/kg every 12 h, 1.96 mg/kg every 12 h, and 2.8 mg/kg every 24 h) (Figure 5) were simulated. A dosage regimen of 1.96 mg/kg every 12 h should be sufficient to reach bactericidal activity in ileum content.

## Discussion

To obtain the best therapeutic effect and to prevent development of resistance in bacteria, the design of dose regiment of antimicrobial agents should be based on their PD and PK properties. Previous PK studies only focused on the plasma concentration of the active substance but ignored the concentrations of antimicrobial agents in their target sites. Innovatively, the pharmacokinetics parameters in both plasma and ileum contents were analyzed in our study.

For the plasma PK study only, the PK parameter (t_1∕2_, *C*_max_, and *T*_max_) obtained in this study were compared with the PK values in previous studies (Pijpers et al., 1997; Richez et al., 1997a,b; Zeng and Fung, 1997; Anadón et al., 1999; Wiuff et al., 2002; Bimazubute et al., 2010) which also treated pigs by IM injection of enrofloxacin at a single dose of 2.5 mg/kg. The *C*_max_ and *T*_max_ obtained in this study were 1.09 μg/mL and 1.27 h, respectively. The range of *C*_max_ and *T*_max_ value obtained in the published literatures were 0.63–1.17 μg/mL and 0.92–1.81 h, respectively. Therefore, the *C*_max_ and *T*_max_ values seem to be in the range of the values obtained previously. However, there seemed some variability between the elimination half-life value in our study (6.69 h) and in other published papers (4.1–13.1 h) (Cox et al., 2004).

As far as we know, other authors have not studied concentrations of enrofloxacin in ileum content. The *ex vivo* ileum cannulation collection devices was firstly used in pigs for our PK/PD study. In ileum content, the values of elimination half-life, *C*_max_, and *T*_max_ were 8.5 h, 7.07 μg/mL, and 5.54 h, respectively. The drug concentration in the intestinal contents greatly exceeded that in chicken plasma (Scheer et al., 1997) and in pig plasma (Lindecrona et al., 2000). The difference in concentrations between plasma and intestinal contents could be because of the high lipophilicity of enrofloxacin. The AUC value showed that enrofloxacin had good penetration to various tissues (Agersø et al., 1998). The ratio of ileum content to plasma (based on AUC values) was 11.3. Enrofloxacin could penetrate membranes and tissues, binding to solid parts of the ileum content. Edlund et al. (1988) showed that up to 95% of the measured norfloxacin concentration in human feces is bound to anaerobic bacteria and solid parts of the feces. The relatively higher binding in humans could be caused by a difference in the composition of feces between humans and pigs.

Enrofloxacin is considered a concentration-dependent agent, and its clinical efficacy is decided by dose and bacterial pathogen. It has been proposed that the break points determining efficacy of fluroquinolone are a *C*_max_/MIC ≥ 10 or an AUC_24h_/MIC ≥ 125 (Rodvold and Neuhauser, 2001). In the present study, the sensitive *E. coli* obtained MIC_50_ and MIC_90_ levels in the ranges of 0.25–0.5 μg/mL, *C*_max_ /MIC, and AUC_24h_/MIC ratios for enrofloxacin in ileum contents were 14.1–28.2 and 272.4–544.7, respectively, indicating that the administration of 2.5 mg/kg enrofloxacin may have a good antibacterial outcome and could be set as an appropriate dose for treatment of *E. coli* infection.

It has been proposed that the AUC_24h_/MPC ratio and T > MPC should be selected as indicators for preventing the drug-resistant mutants (Zhao and Drlica, 2001; Olofsson et al., 2006). The AUC_24h_/MPC in our study (18.8 h) was more than that obtained in the previous studies (9.7 h) conducted for enrofloxacin against *E. coli* isolates of dog origin (Gebru et al., 2011), and the *C*_max_/MPC ratio (0.99) calculated in our study was close to that obtained in previous reports (1.4) for enrofloxacin against *E. coli* (Gebru et al., 2011). Previous study demonstrated that ciprofloxacin treatment at AUC/MPC ratios of 22 could prevent the resistant mutants of *E. coli* with an inoculum sizes of 10^10^ cfu/mL (Olofsson et al., 2006).

A number of studies have found that the increasing level of antimicrobial resistance in gut flora was mainly due to the misuse of antimicrobial agents in unsuitable and/or irrespective administration route (Nguyen et al., 2012; Zhang et al., 2013). It is very important to optimize the dose regiment by considering both the PK and PD parameters of antimicrobial agents. The PK/PD combined model can provide dose optimization and combine the relationship between *in vitro* and *in vivo* study. According to our PK and PD parameters, the single dose required to reach bacteriostatic, bactericidal and eradication activity were 0.78, 1.96, and 2.8 mg/kg, respectively. After simulating different dosage regimens, it was found that an every 12 h treatment of 1.96 mg/kg enrofloxacin for 3 days could sufficiently clear *E. coli* infection in pigs. Our model is depend on bacterial population and PK parameters obtained from healthy animals. It may be more wise to obtain more information about the population pharmacokinetics of enrofloxacin to take into account all the variability associated with animals and bacteria.

## Conclusion

The purpose of our study was to estimate a dosage regimen of enrofloxacin by IM administration that would be sufficient for treatment of *E. coli* in pigs. The dosage regimen was designed on a PK analysis and some PD studies in ileum contents. PD parameters of enrofloxacin were derived from the analysis of static time kill curves obtained *ex vivo*. The dosage regimen was simulated using an *E*_max_ model. As a concentration-dependent drug, this approach for dose determination is better than the analysis of the relationship between AUC/MIC value and the number of viable bacteria after 24 h. A dosage regimen of 1.96 mg/kg every 12 h for 3-days should be sufficient in the treatment of *E. coli*.

### Conflict of interest statement

The authors declare that the research was conducted in the absence of any commercial or financial relationships that could be construed as a potential conflict of interest.
